# An Impedance Readout IC with Ratio-Based Measurement Techniques for Electrical Impedance Spectroscopy

**DOI:** 10.3390/s22041563

**Published:** 2022-02-17

**Authors:** Song-I Cheon, Soon-Jae Kweon, Youngin Kim, Jimin Koo, Sohmyung Ha, Minkyu Je

**Affiliations:** 1 School of Electrical Engineering, Korea Advanced Institute of Science and Technology (KAIST), Daejeon 34141, Korea; 1000thd2@kaist.ac.kr (S.-I.C.); youngin@kaist.ac.kr (Y.K.); jimin_koo@kaist.ac.kr (J.K.); 2 Division of Engineering, New York University Abu Dhabi, Abu Dhabi 129188, United Arab Emirates; sj.kweon89@nyu.edu; 3 Tandon School of Engineering, New York University, New York, NY 10003, USA

**Keywords:** electrical impedance spectroscopy, bioimpedance, real/magnitude measurement, demodulator, ratio-based detection, low-complexity design

## Abstract

This paper presents an error-tolerant and power-efficient impedance measurement scheme for bioimpedance acquisition. The proposed architecture measures the magnitude and the real part of the target complex impedance, unlike other impedance measurement architectures measuring either the real/imaginary components or the magnitude and phase. The phase information of the target impedance is obtained by using the ratio between the magnitude and the real components. This can allow for avoiding direct phase measurements, which require fast, power-hungry circuit blocks. A reference resistor is connected in series with the target impedance to compensate for the errors caused by the delay in the sinusoidal signal generator and the amplifier at the front. Moreover, an additional magnitude measurement path is connected to the reference resistor to cancel out the nonlinearity of the proposed system and enhance the settling speed of the low-pass filter by a ratio-based detection. Thanks to this ratio-based detection, the accuracy is enhanced by 30%, and the settling time is improved by 87.7% compared to the conventional single-path detection. The proposed integrated circuit consumes only 513 μW for a wide frequency range of 10 Hz to 1 MHz, with the maximum magnitude and phase errors of 0.3% and 2.1°, respectively.

## 1. Introduction

Electrical or electrochemical impedance spectroscopy (EIS) is an analytical technique measuring the target impedance over a frequency range. It is widely used in a variety of applications, including ischemia detection [1,2,3], glucose sensing [4], lung monitoring [5,6], DNA analysis [7,8], common allergens detection [9], food pathogen detection [10], and battery monitoring [11]. This technique also has been applied to the early detection of cancers such as cervical cancer [12], prostate cancer [13,14], skin cancer [15], colorectal cancer [16], and breast cancer [17,18]. Since EIS allows for noninvasive, low-cost, effective monitoring of the target impedance with a small form factor [19,20,21], wearable implementation for the aforementioned applications would be greatly beneficial. For that purpose, it is important to design a robust, low-power circuit architecture for impedance measurements.

**Figure 1:**
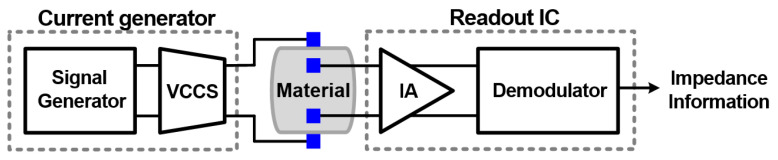
Overall block diagram of an EIS system.

Figure 1 shows an overall block diagram of the EIS system composed of a current generator and a readout integrated circuit (IC). The current generator typically consists of a signal generator, which creates a sinusoidal voltage waveform with variable frequencies, and a voltage-controlled current source (VCCS), which converts the voltage waveform into a current [22,23]. The current is applied across the target material, which has a complex impedance. The measurement IC typically amplifies the measured voltage signal across the target material by an instrumentation amplifier (IA) and then uses its demodulator to obtain the impedance information.

**Figure 2:**
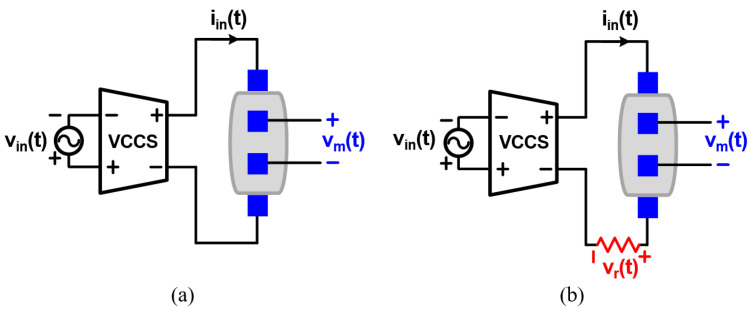
Impedance measurement structures (**a**) without and (**b**) with a reference resistor connected in series with the target impedance.

Figure 2 shows two impedance measurement structures with and without a reference resistor, respectively. In both structures, the VCCS transforms the input voltage signal, $v_{\mathrm{in}}\left(t\right)$, to the output current signal, $i_{\mathrm{in}}\left(t\right)$. Then, $i_{\mathrm{in}}\left(t\right)$ is applied to the material, and the resulting voltage, $V_{m}\left(t\right)$, is measured. In the most basic structure shown in Figure 2a, $i_{\mathrm{in}}\left(t\right)$ flows through the target impedance only. Inherently, the VCCS incurs delay and nonlinearity on $i_{\mathrm{in}}\left(t\right)$, resulting in an error in the final measurement result. In order to solve this problem, a reference resistor can be added in series with the target impedance, as shown in Figure 2b ([24]). The same $i_{\mathrm{in}}\left(t\right)$ flows through both the target impedance and reference resistor together, generating two voltage outputs, $v_{m}\left(t\right)$ and $v_{r}\left(t\right)$. Since the impedance of resistors has a zero phase, the voltage signal across the reference resistor, $v_{r}\left(t\right)$, has the same phase as $i_{\mathrm{in}}\left(t\right)$. Thus, $v_{r}\left(t\right)$ can serve as a reference of the phase and magnitude measurements, so the effects from the delay or nonlinearity of the VCCS can be avoided.

**Figure 3:**
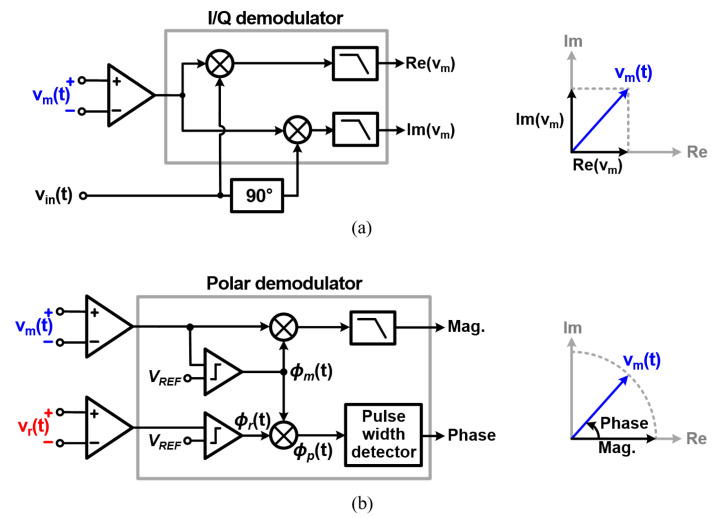
Representative structures of (**a**) the I/Q demodulator and (**b**) polar demodulator.

Despite the advantages of the reference resistor, the conventional I/Q demodulators, which require quadrature signal generation, are mostly based on the structure of Figure 2a [17,20,25,26,27]. As shown in Figure 3a, the I/Q demodulator structure obtains the real component of the target impedance by mixing the signal from the target impedance with $v_{\mathrm{in}}\left(t\right)$ and the imaginary component by mixing it with a signal that is 90°-phase-shifted from $v_{\mathrm{in}}\left(t\right)$ [17,20,25,26,27]. Since only the signal corresponding to the frequency of $v_{\mathrm{in}}\left(t\right)$ is converted to the DC value, it is insensitive to noises at other frequencies. However, it requires the 90°-shifted (quadrature) signal for variable frequencies. In order to utilize the reference resistor, it is necessary to delay the signal from the reference resistor by the time interval corresponding to 90° of phase for variable frequencies in the demodulator. So, additional complex techniques with considerable hardware and power overhead should be used, such as an oversampling mixer employing an eight-phase local oscillator [28], direct digital synthesis (DDS) block [29], a large-area passive network [30], or an edge detector, which requires a 50-times-faster clock [17]. To avoid this complexity, the I/Q demodulator receives the 90°-shifted signal from the current generator instead of the reference resistor. However, this makes the structure vulnerable to the phase error between $v_{\mathrm{in}}\left(t\right)$ and the IA output caused by the delay of the VCCS and IA. Therefore, for high measurement accuracy, a phase-correction circuit is needed [31,32], inducing another power overhead and design complexity.

On the other hand, the polar demodulator structure is suitable for using the reference resistor. It measures the magnitude and phase components by using the measurements of both $v_{m}\left(t\right)$ and $v_{r}\left(t\right)$. Various magnitude measurement techniques have been demonstrated, such as adaptive sampling [32,33], peak detecting [34], and time-stamping schemes [35]. Among them, one of the most commonly used methods is the one based on a self-mixing full-wave rectifier, as shown in Figure 3b [24]. Since it does not suffer from the delays of the amplifier and VCCS, this method is robust over a wide frequency range compared to the other structures.

**Figure 4:**
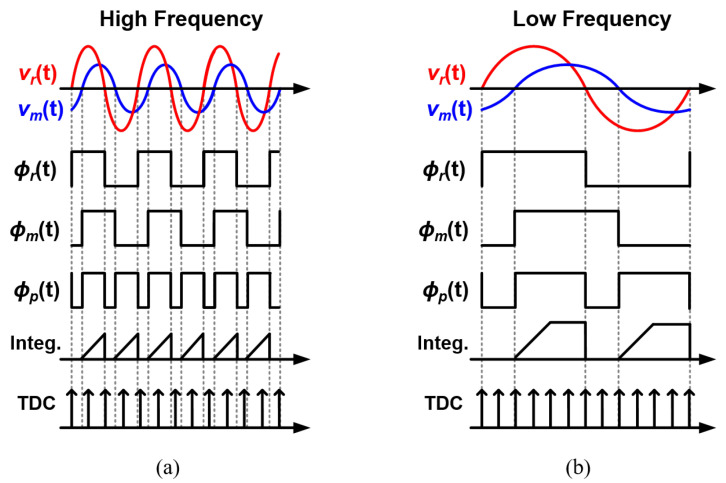
Conceptual representation of the phase-detection process (**a**) at high frequency and (**b**) low frequency.

On the contrary, the phase measurement in the polar structure typically requires complex and power-hungry circuit blocks to cover a wide frequency range [24,32,33,34]. Figure 4 shows the phase-detection process when using a time-to-digital converter (TDC) or an integrator to measure the phase difference between $v_{m}\left(t\right)$ and $v_{r}\left(t\right)$. $\phi_{r}\left(t\right)$ and $\phi_{m}\left(t\right)$ are the zero-crossing comparison results of $v_{r}\left(t\right)$ and $v_{m}\left(t\right)$, respectively. By mixing $\phi_{r}\left(t\right)$ and $\phi_{m}\left(t\right)$, typically using an XNOR gate, $\phi_{p}\left(t\right)$ is created. Then, the duty ratio of $\phi_{p}\left(t\right)$ corresponds to the phase difference, and the pulse width of $\phi_{p}\left(t\right)$ should be quantified. Since the pulse width of $\phi_{p}\left(t\right)$ varies inversely proportional to the input frequency for the same phase difference, the comparator must cover a wide frequency range. When an integrator is used to measure the pulse width, it must quickly integrate $\phi_{p}\left(t\right)$ with a large current to support high frequencies [36,37]. This fast integration, however, causes saturation of the integrator output at low frequencies, as shown in Figure 4b. In order to cover a wide frequency range without saturation, it is necessary to use a high-resolution analog-to-digital converter, resulting in high power consumption. When a TDC is used to measure the pulse width, it requires a high-speed clock to operate at high frequencies, thus increasing power consumption.

Alternatively, a low-pass filter (LPF) can be used to reduce power consumption and avoid design complexity [38]. It creates a DC value by averaging the signal $\phi_{p}\left(t\right)$ regardless of the frequency. The DC output is proportional to the pulse width and hence to the phase of the target impedance. Due to its simplicity, this has been widely used not only for the phase measurement in this structure but also for the real, imaginary, and magnitude measurements in various architectures. Although the LPF can easily support a wide frequency range with low power consumption, it suffers from a long settling time, significantly slowing down the measurement speed.

In this paper, we present an impedance readout IC that measures the real part and magnitude of the target impedance using a ratio-based detection technique. The proposed IC is based on the polar demodulator with the reference resistor, making the system robust to the errors originating from nonidealities of the VCCS. Whereas other polar demodulators measure the magnitude and phase, inherently suffering from complex circuits, high power consumption, and/or the throughput problem, the proposed one measures the magnitude and real component instead. The magnitude is obtained using a self-mixing full-wave rectifier to make it less susceptible to the delay of the IA. For the real-component measurement, another mixing process is performed in the magnitude measurement path. In this process, the signal from the target impedance is mixed with the signal from the comparator and IA connected to the reference resistor. Because the same IA is shared in this path, the IA’s nonlinearity can be cancelled out. This also relaxes the bandwidth requirement of the IA, reducing the overall power consumption. Moreover, this architecture allows for obtaining the final impedance result by using the ratio of the results obtained in the paths, significantly improving the robustness and throughput. As a result, the proposed IC can easily achieve low power consumption and support a wide frequency range with low circuit complexity while providing fast settling.

The rest of the paper is organized as follows. The overall architecture of the proposed IC is explained in Section 2, and the detailed implementation is described in Section 3. Section 4 shows the simulation results, and Section 5 concludes the paper.

## 2. Proposed Architecture

**Figure 5:**
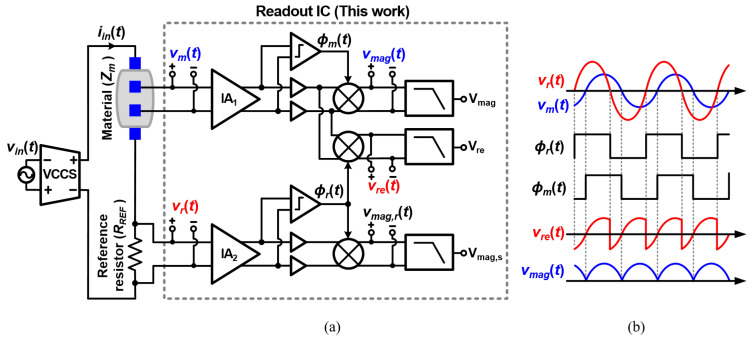
(**a**) Simplified block diagram of the proposed impedance measurement IC and (**b**) its operation waveforms.

Figure 5a shows the overall architecture of the proposed impedance readout IC consisting of two IAs, three passive mixers, two comparators and three LPFs to produce three final outputs ($V_{mag}$, $V_{re}$, and $V_{mag,r}$). These circuit blocks form two magnitude measurement paths (Mag-paths) for $V_{mag}$ and $V_{mag,r}$ and one real-component measurement path (Real-path) for $V_{re}$. The sinusoidal input current signal ($i_{in}\left(t\right)$), which is driven by the VCCS, flows through the target impedance ($Z_{m}$) and reference resistor ($R_{REF}$). The voltage signals across $Z_{m}$ and $R_{REF}$ ($v_{m}\left(t\right)$ and $v_{r}\left(t\right)$, respectively) are applied to the input of the readout IC. These voltages are expressed as:

(1) $$\begin{array}{cc} v_{m}\left(t\right) & =|Z_{m}|A_{in}cos\left(2\pi f_{in}t+\theta_{VCCS}+\theta_{m}\right)\\ v_{r}\left(t\right) & =R_{REF}A_{in}cos\left(2\pi f_{in}t+\theta_{VCCS}\right) \end{array}$$

where $A_{in}$ is the amplitude of the IA, $f_{in}$ the frequency of $i_{in}\left(t\right)$, $\theta_{m}$ the phase of $Z_{m}$, and $\theta_{VCCS}$ the phase error in the VCCS. These two signals are amplified by the two identical IAs and converted into square-wave signals, $\phi_{m}\left(t\right)$ and $\phi_{r}\left(t\right)$, by two zero-crossing comparators, as shown in Figure 5b. $\phi_{m}\left(t\right)$ and $\phi_{r}\left(t\right)$ correspond to the phase information of each signal. The amplified $v_{m}\left(t\right)$ and $v_{r}\left(t\right)$ pass through buffers, which replicate the delay in $\phi_{m}\left(t\right)$ and $\phi_{r}\left(t\right)$ by the comparators. These sinusoidal signals are mixed with $\phi_{m}\left(t\right)$ and $\phi_{r}\left(t\right)$ in the mixers. The buffers also reduce the kick-back noise from the mixer output to the comparator and IA. The resulting voltage signals from the demodulation, $v_{mag}\left(t\right)$, $v_{re}\left(t\right)$, and $v_{mag,r}\left(t\right)$, go through LPFs, generating the DC voltages, $V_{mag}$, $V_{re}$, and $V_{mag,r}$. These DC outputs represent the magnitude and real part of the target impedance, and the magnitude of the reference resistor, respectively.

### 2.1. Magnitude Measurement Path

The magnitude information from $Z_{m}$ and $R_{REF}$ is extracted by two Mag-paths, composed of identical circuit components, as shown in Figure 5a. After $v_{m}\left(t\right)$ is amplified, the signal is processed by the comparator, producing $\phi_{m}\left(t\right)$, which periodically repeats the values of $V_{DD}$ (= 1.8 V) and 0 V. A chopper switches the polarity of $v_{m}\left(t\right)$ when $\phi_{m}\left(t\right)$ toggles. As a result, the chopper mixes the amplified $v_{m}\left(t\right)$ and $\phi_{m}\left(t\right)$, generating $v_{mag}\left(t\right)$. The rectified signal, $v_{mag}\left(t\right)$, can be expressed as follows:

(2) $$\begin{array}{cc} v_{mag}\left(t\right) & =A_{IA1}v_{m}\left(t\right)\times \phi_{m}\left(t\right)\\ & =|Z_{m}|A_{in}A_{IA_{1}}cos\left(2\pi f_{in}t+\theta_{VCCS}+\theta_{{IA}_{1}}+\theta_{B_{1}}+\theta_{m}\right)\times cos\left(2\pi f_{in}t+\theta_{VCCS}+\theta_{{IA}_{1}}+\theta_{C_{1}}+\theta_{m}\right)\\ & =\frac{|Z_{m}|A_{in}A_{{IA}_{1}}}{2}\left[cos\left(\theta_{C_{1}}-\theta_{B_{1}}\right)+cos\left(4\pi f_{in}t+2\theta_{VCCS}+2\theta_{{IA}_{1}}+\theta_{B_{1}}+\theta_{C_{1}}+2\theta_{m}\right)\right] \end{array}$$

where $\theta_{{IA}_{1}}$, $\theta_{B_{1}}$, and $\theta_{C_{1}}$ are the phase delays of the IA${}_{1}$, buffer, and comparator in the $Z_{m}$ Mag-path, respectively. The equation does not include the mixing results between $v_{m}\left(t\right)$ and the harmonics of $\phi_{m}\left(t\right)$ because they are located at high frequencies and will be filtered out easily by the LPF. In addition, the second term located at the second harmonic frequency ($cos(4\pi f_{in}t+2\theta_{VCCS}+2\theta_{IA1}+\theta_{B}+\theta_{C1}+2\theta_{m})$) is filtered out by the subsequent LPF. Then, the DC term of $|Z_{m}|A_{in}A_{IA1}cos\left(\theta_{C1}-\theta_{B1}\right)/2$ only remains after being multiplied by 2/π. Thus, the final output is a DC value expressed as:

(3) $$\begin{array}{c} V_{mag}=\frac{|Z_{m}|A_{in}A_{{IA}_{1}}}{\pi }cos\left(\theta_{C_{1}}-\theta_{B_{1}}\right) \end{array}$$

Note that $V_{mag}$ does not include the error terms related to $\theta_{VCCS}$ and $\theta_{IA1}$, which are cancelled out during the mixing operation. In addition, by adding the buffers between the IA and mixer and making $\theta_{C_{1}}$ and $\theta_{B_{1}}$ similar, the error caused by the comparator delay can be compensated. Now $cos(\theta_{C1}-\theta_{B1})\approx 1$, $V_{mag}$ becomes

(4) $$\begin{array}{c} V_{mag}\approx \frac{|Z_{m}|A_{in}A_{{IA}_{1}}}{\pi } \end{array}$$

The magnitude value of the target impedance, $|Z_{m}|$, can be found as:

(5) $$\begin{array}{c} |Z_{m}|=\frac{\pi V_{mag}}{A_{in}A_{{IA}_{1}}cos\left(\theta_{C_{1}}-\theta_{B_{1}}\right)}\approx \frac{\pi V_{mag}}{A_{in}A_{{IA}_{1}}} \end{array}$$

Note that $|Z_{m}|$ is not affected by phase delays of any circuit components. The equation still includes $A_{in}$ and $A_{{IA}_{1}}$, so $|Z_{m}|$ is affected by the nonlinearity and frequency response of the VCCS and IA like many other conventional impedance measurement circuits.

In our work, another Mag-path is added to achieve even higher accuracy and robustness. The $R_{REF}$ Mag-path is added to compensate for the nonlinearity of the VCCS and IA. In this path, the final output is produced using the same process as the $Z_{m}$ Mag-path. Then, $V_{mag,r}$ is given as:

(6) $$\begin{array}{c} V_{mag,r}=\frac{R_{REF}A_{in}A_{{IA}_{2}}}{\pi }cos\left(\theta_{C_{2}}-\theta_{B_{2}}\right)\approx \frac{R_{REF}A_{in}A_{{IA}_{2}}}{\pi } \end{array}$$

where $\theta_{B_{2}}$ and $\theta_{C_{2}}$ are the delays of the buffer and comparator in the $R_{REF}$ Mag-path, respectively. The final magnitude result, MAG($Z_{m}$), is calculated by taking the ratio between the outputs from the two Mag-paths, which are described by Equations (3) and (6).

(7) $$\begin{array}{cc} MAG(Z_{m}) & =\frac{V_{mag}}{V_{mag,r}}\times R_{REF} \end{array}$$

The error between the final magnitude result and actual impedance magnitude $|Z_{m}|$ can be found from the following equation:

(8) $$\begin{array}{cc} MAG(Z_{m}) & =\frac{V_{mag}}{V_{mag,r}}\times R_{REF}\\ & =\frac{A_{in}A_{{IA}_{1}}\left|Z_{m}\right|cos\left(\theta_{C_{1}}-\theta_{B_{1}}\right)/2}{A_{in}A_{{IA}_{2}}R_{REF}cos\left(\theta_{C_{2}}-\theta_{B_{2}}\right)/2}\times R_{REF}\\ & =\frac{A_{{IA}_{1}}}{A_{{IA}_{2}}}\times \frac{cos(\theta_{C_{1}}-\theta_{B_{1}})}{cos(\theta_{C_{2}}-\theta_{B_{2}})}\times \left|Z_{m}\right| \end{array}$$

The error factor between the measured value (MAG($Z_{m}$)) and the actual value ($|Z_{m}|$) is $\frac{A_{{IA}_{1}}}{A_{{IA}_{2}}}\times \frac{cos(\theta_{C_{1}}-\theta_{B_{1}})}{cos(\theta_{C_{2}}-\theta_{B_{2}})}$, which is composed of ratios between circuit parameters of the two Mag-paths. It indicates that the magnitude measurement accuracy is only affected by the mismatch between the two Mag-paths instead of the nonlinearity and phase delays of the circuit components such as the VCCS, IAs, and comparators. Thanks to this ratio-based method, the proposed readout circuit becomes much less susceptible to the nonlinearity and gain-bandwidth variation of the VCCS and IA. In addition, the settling slope of the LPF output is similar in the two Mag-paths, so the ratio between $V_{mag}$ and $V_{mag,r}$ settles much faster than $V_{mag}$ or $V_{mag,r}$ themselves. This characteristic is explained and demonstrated in Section 4.

### 2.2. Real-Component Measurement Path

The real component of the target impedance can be obtained by demodulating $v_{m}\left(t\right)$ using $\phi_{r}\left(t\right)$, which is derived from the reference resistor $R_{REF}$, as shown in Figure 5a. Assuming that the amplitude of the fundamental tone of $\phi_{r}\left(t\right)$ is 1, the demodulated real component signal ($v_{re}\left(t\right)$) is expressed as:

(9) $$\begin{array}{cc} v_{re}\left(t\right) & =|Z_{m}|A_{in}A_{{IA}_{1}}cos\left(2\pi f_{in}t+\theta_{VCCS}+\theta_{{IA}_{1}}+\theta_{B_{1}}+\theta_{m}\right)\times cos\left(2\pi f_{in}t+\theta_{VCCS}+\theta_{{IA}_{2}}+\theta_{C_{2}}\right)\\ & =\frac{|Z_{m}|A_{in}A_{IA1}}{2}\left[cos\left(\theta_{m}+\theta_{{IA}_{1}}-\theta_{{IA}_{2}}+\theta_{B1}-\theta_{C_{2}}\right)\right)\\ & +cos(4\pi f_{in}t+2\theta_{VCCS}+\theta_{{IA}_{1}}+\theta_{{IA}_{2}}+\theta_{B_{1}}+\theta_{C_{2}}+\theta_{m})] \end{array}$$

where $\theta_{IA2}$ is the phase delay of IA${}_{2}$. In this equation, $\phi_{r}\left(t\right)$ is assumed to be a pure-tone cosine waveform having the fundamental tone only, as in Equation (2). This is because the mixing results between $v_{m}\left(t\right)$ and the harmonics of $\phi_{m}\left(t\right)$ are located at high frequencies and will be filtered out easily by the subsequent LPF. By low-pass filtering, the second term at $2f_{in}$ is filtered out, and only the DC term is left as:

(10) $$\begin{array}{c} V_{re}=\frac{|Z_{m}|A_{in}A_{{IA}_{1}}}{\pi }cos\left(\theta_{m}+\theta_{{IA}_{1}}-\theta_{{IA}_{2}}+\theta_{B1}-\theta_{C_{2}}\right) \end{array}$$

As shown in Equations (9) and (10), the VCCS delay is cancelled out by the mixing operation. In addition, the IA delays ($\theta_{{IA}_{1}}$ and $\theta_{{IA}_{2}}$) can also be cancelled out because IA${}_{1}$ and IA${}_{2}$ are identical, and their delays are close to each other. Thus, $\theta_{{IA}_{1}}$ and $\theta_{{IA}_{2}}$ are almost completely cancelled out each other as follows:

(11) $$\begin{array}{c} V_{re}\approx \frac{|Z_{m}|A_{in}A_{{IA}_{1}}}{\pi }cos\left(\theta_{m}+\theta_{B1}-\theta_{C_{2}}\right) \end{array}$$

The phase error term ($\theta_{B_{1}}-\theta_{C_{2}}$) is also very small compared to $\theta_{m}$ because the buffer is added to match the delay of the comparator. Thus, the equation can be written as:

(12) $$\begin{array}{cc} V_{re} & \approx \frac{|Z_{m}|A_{in}A_{{IA}_{1}}}{\pi }cos\left(\theta_{m}\right)\\ & =Re\left[Z_{m}\right]\times \frac{A_{in}A_{{IA}_{1}}}{\pi } \end{array}$$

Based on this equation, $Re[Z_{m}]$ can be found as:

(13) $$\begin{array}{c} Re\left[Z_{m}\right]=\frac{\pi V_{re}}{A_{in}A_{{IA}_{1}}} \end{array}$$

Similar to Equation (5), this result is prone to gain errors in the VCCS and IA because their gain terms are included in the calculation.

To address this issue, the final real-component result, $RE(Z_{m})$, can be obtained by using a ratio, as in Equation (7):

(14) $$\begin{array}{cc} RE(Z_{m}) & =\frac{V_{re}}{V_{mag,r}}\times R_{REF} \end{array}$$

As such, $RE(Z_{m})$ is calculated from the ratio between the two DC voltage outputs, multiplied by $R_{REF}$. The deviation of $RE(Z_{m})$ from $Re[Z_{m}]$ can be performed as follows:

(15) $$\begin{array}{cc} RE(Z_{m}) & =\frac{V_{re}}{V_{mag,r}}\times R_{REF}=\frac{A_{in}A_{{IA}_{1}}\left|Z_{m}\right|cos\left(\theta_{m}+\theta_{e}\right)/\pi }{A_{in}A_{{IA}_{2}}R_{REF}/\pi }\times R_{REF}\\ & =\frac{A_{{IA}_{1}}}{A_{{IA}_{2}}}\times \left|Z_{m}\right|cos\left(\theta_{m}+\theta_{e}\right)\\ & \approx |Z_{m}|cos\left(\theta_{m}\right)=Re\left[Z_{m}\right] \end{array}$$

where $\theta_{e}=\theta_{B_{1}}-\theta_{C_{2}}$. As shown in Equation (15), the effects from the nonlinearity and delay of the VCCS and IA are canceled out.

The final phase measurement result, $PHASE(Z_{m})$, can also be obtained from a ratio between the voltage outputs as follows:

(16) $$\begin{array}{cc} cos(PHASE\left(Z_{m}\right)) & =\frac{V_{re}}{V_{mag}}=\frac{A_{in}A_{{IA}_{1}}\left|Z_{m}\right|cos\left(\theta_{m}+\theta_{e}\right)/\pi }{A_{in}A_{{IA}_{1}}\left|Z_{m}\right|cos\left(\theta_{e}\right)/\pi }\\ & =\frac{cos(\theta_{m}+\theta_{e})}{cos(\theta_{e})}\approx cos\left(\theta_{m}\right)\\ PHASE(Z_{m}) & \approx \theta_{m} \end{array}$$

As shown in the equation, the phase output is affected by $\theta_{e}$, which is reduced by matching the comparator and buffer delays.

In the proposed impedance readout IC, the magnitude, real component, and phase of the target impedance are measured using ratio-based techniques. Thanks to this, the results are robust to the variations in circuit parameters. Moreover, the IAs do not need to have too wide bandwidths. The gain degradation of the IA at high frequencies is also compensated so that the IA can be designed to consume lower power.

## 3. Circuit Implementation

### 3.1. Instrumentation Amplifier

**Figure 6:**
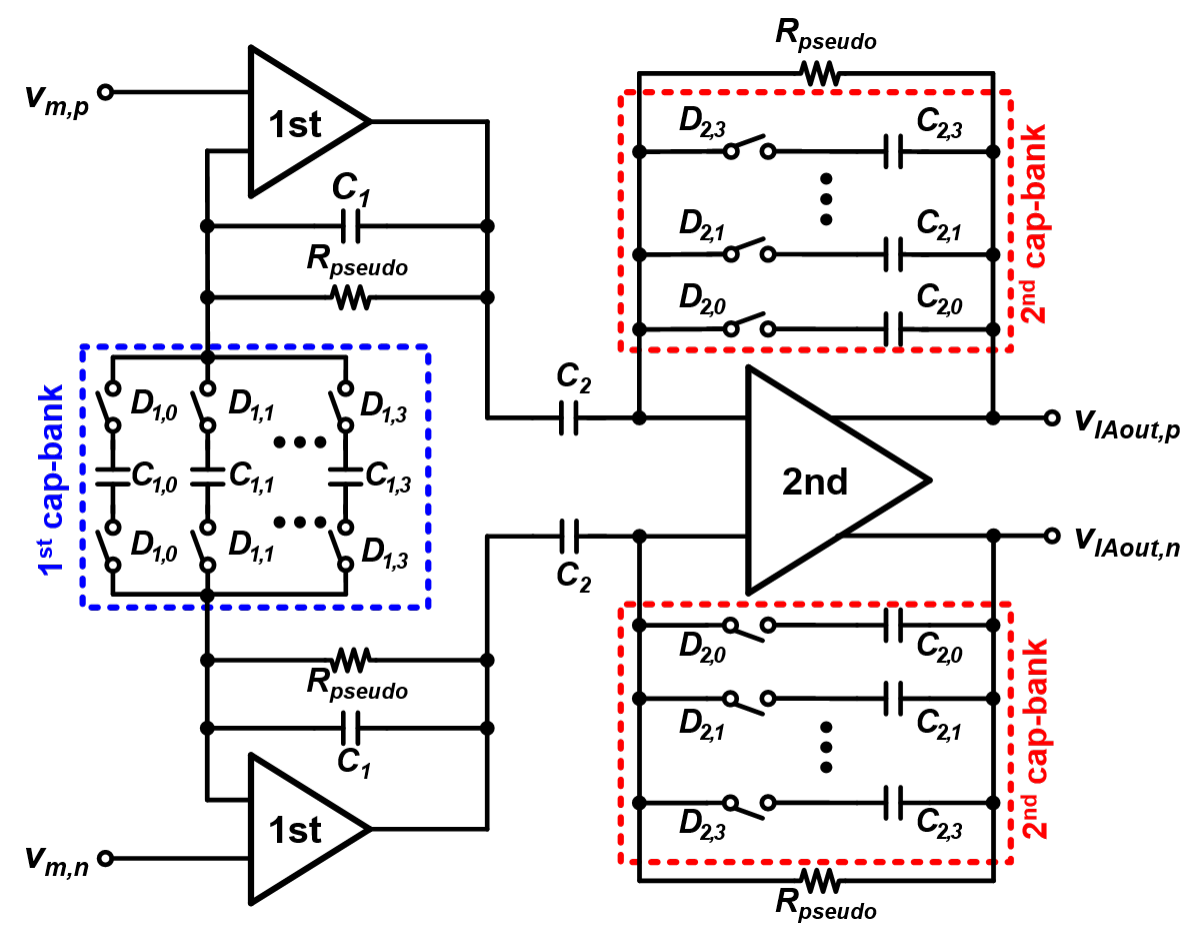
Circuit schematic of the IA.

As shown in Figure 6, the IAs consist of three op-amps, resistors, and capacitors including three reconfigurable capacitor banks (cap-banks). The first stage of the IA is composed of two differential-to-single-ended amplifiers while a fully differential amplifier is used for the second stage. Each cap-bank contains capacitors and switches, which are controlled by 4-bit digital words. By changing these capacitor values, the IA gain can be configured from 7 to 48.7 dB. The bandwidth of the IA is 225 kHz at the maximum gain mode, with gains of 41.4 dB and 41.9 dB at 10 Hz and 1 MHz, respectively. In the minimum gain mode, it has a bandwidth of 3.6 MHz, and the gains at 10 Hz and 1 MHz are 6.95 dB and 6.9 dB, respectively. Since the impedance measurement is performed by using the ratio between the magnitude and real values of the target impedance and $R_{REF}$, even the 225-kHz IA bandwidth is sufficient to cover the 1 MHz input frequency range.

**Figure 7:**
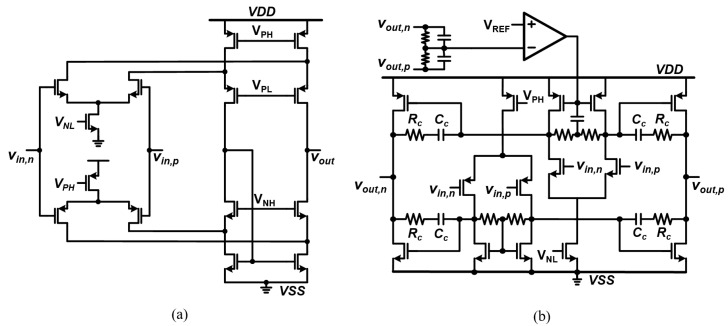
Circuit schematic of (**a**) the first-stage amplifier and (**b**) the second-stage amplifier.

Figure 7 shows the detailed schematic diagrams of the first- and second-stage amplifiers. The first-stage amplifier is designed as a rail-to-rail folded-cascode amplifier to provide a wide input range and excellent stability when driving a large capacitor $C_{2}$ as a load. The two-stage op-amp shown in Figure 7b is used as the second-stage amplifier to support a large output swing range. It also employs a common-mode feedback to maintain the common-mode voltage stably.

### 3.2. Comparator

**Figure 8:**
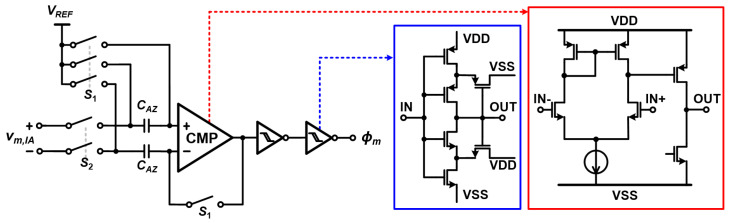
Circuit schematic of the comparator.

The comparator is implemented with the autozeroing scheme to remove the offset, as shown in Figure 8. To enhance the effect of the autozeroing technique, the comparator is designed as a single amplifier stage instead of cascading multiple stages of amplifiers. In addition, two Schmitt triggers are added after the comparator to make a glitch-free rectangular waveform. Two capacitors ($C_{\mathrm{AZ}}$) and six switches are used for the autozeroing technique. In the first phase, the four $S_{1}$ switches are closed and the two $S_{2}$ switches are open. Then, the comparator offset is stored in the $C_{\mathrm{AZ}}$ placed at the negative input of the comparator. This stored offset is used to cancel out the effect from the offset in the next phase when the $S_{2}$ switches are closed and the $S_{1}$ switches are open. Since the offset stored in $C_{\mathrm{AZ}}$ discharges over time, it is important to perform the autozeroing periodically. The autozeroing can be performed in between each impedance measurement for different frequencies.

**Figure 9:**
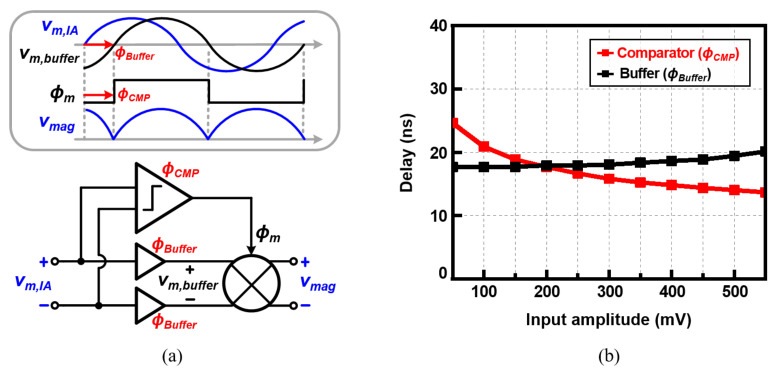
(**a**) Block diagram and operation waveforms of the mixing part of the proposed work, and (**b**) simulated input–output delays of the comparator and the buffer over the input amplitude when the input frequency is set to 1 MHz.

Figure 9a shows a block diagram of the part, including the chopper, comparator, and buffers, as well as a timing diagram of the main signals. When the IA output signal ($v_{m,IA}$) passes through the comparator and generates the square-wave signal ($\phi_{m}$), the signal is affected by the comparator delay ($\phi_{CMP}$). The comparator output after the delay is $\phi_{m}$. Thanks to the buffer, this delay is canceled out by the buffer delay ($\phi_{Buffer}$). Figure 9b compares the delay of the comparator and the buffer as a function of the input amplitude. The comparator’s delay varies over the input amplitude. As shown in the figure, the delay difference between the comparator and buffer is up to 7 ns, which is much smaller than the delay of the comparator of 14–25 ns. This reduces the phase error by more than 50%. To maximize the delay cancellation effect, the buffer is designed to have an average delay of the comparator delay. If the amplitude of the comparator input is 100–400 mV by configuring the gain of IA, the error is reduced to within 3.8 ns.

### 3.3. Low-Pass Filter

**Figure 10:**
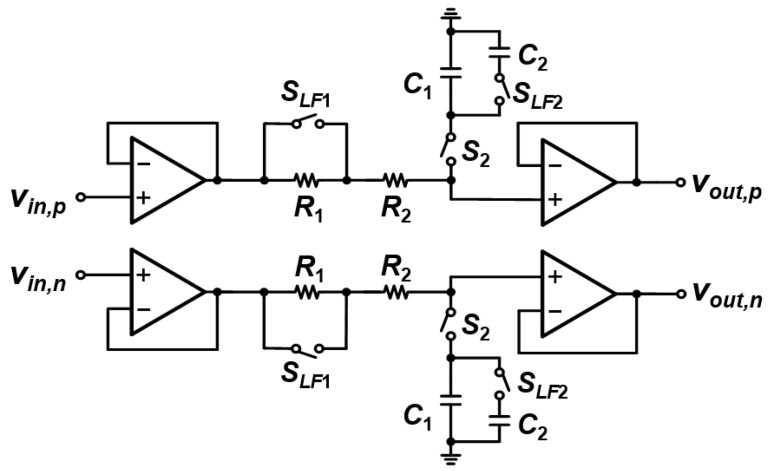
Circuit diagram of the LPF.

Figure 10 shows the schematic of the LPF. Four buffers are used to isolate the input and output signals, and they are implemented as the folded-cascode amplifier structure. The cut-off frequency of the LPF can be controlled by $S_{\mathrm{LF}1}$ and $S_{\mathrm{LF}2}$ switches to speed up the measurement time and reduce the ripple by sufficiently attenuating the high-frequency terms. $R_{1}$, which has 10-times-greater resistance than $R_{2}$, and $C_{2}$, which has 100-times-greater capacitance than $C_{1}$, are used with these switches. There is also an additional switch, $S_{2}$, which operates with the same clock for the autozeroing, to avoid additional settling time that occurs when $C_{\mathrm{AZ}}$ stores the offset voltage.

## 4. Results

The proposed IC was designed in a TSMC 180 nm complementary metal-oxide- semiconductor (CMOS) process. As shown in Figure 5, simulations were performed by applying a sinusoidal current with the same amplitude (4 μA) to the target impedance and reference resistor for 11 points in the frequency range from 10 Hz to 1 MHz. The target impedance is based on a simplified cell model, which is composed of a 200 Ω resistor in series with a parallel combination of a 45 nF capacitor and a 5 kΩ resistor [24]. The $R_{REF}$ values of 500 Ω and 5 kΩ are used for the frequency ranges of 5 kHz–1 MHz and 10 Hz–5 kHz, respectively. The LPF cut-off frequency was adjusted so that the ripple was small enough for the applied frequency, and the IA gain was chosen according to the output DC value.

### 4.1. IA and LPF

**Figure 11:**
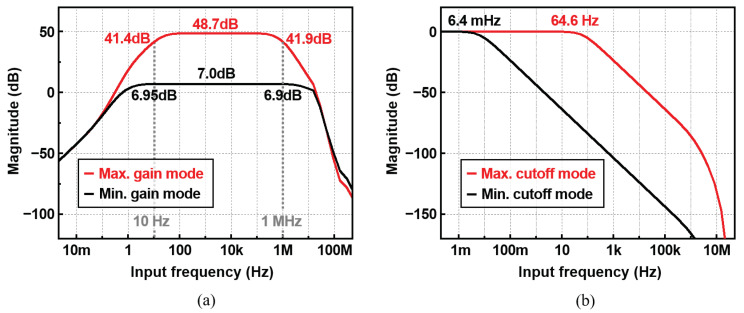
Frequency response of (**a**) the IA and (**b**) the LPF.

Figure 11a,b shows the frequency response of the IA and the LPF. The IA covers the frequency from 30 Hz to 225 kHz for the maximum gain mode, and the frequency from 7 Hz to 3.7 MHz for the minimum gain mode, as shown in Figure 11a. In the simulation conducted using the impedance model, a gain mode of 33 dB was used up to 5 kHz, and a maximum gain mode was used for frequencies above that. Figure 11b plots the cut-off frequency variation of LPF. The maximum and minimum cut-off frequencies of the LPF are 64.6 Hz and 6.4 mHz, respectively. In order to remove the second-order harmonic components at $2f_{\mathrm{in}}$, which ranges from 20 Hz to 2 MHz, the cut-off frequency can be set to be low enough. The cut-off frequencies of 6.4 mHz, 64 mHz, 640 mHz, 6.4 Hz, and 64.6 Hz are used for the frequency ranges of 10–50 Hz, 50–500 Hz, 500 Hz–5 kHz, and 5 kHz–1 MHz, respectively. When the lowest input signal (10 Hz) is applied, the LPF attenuates the 20 Hz signal around 65 dB.

### 4.2. Chopper and Comparator Operation

**Figure 12:**
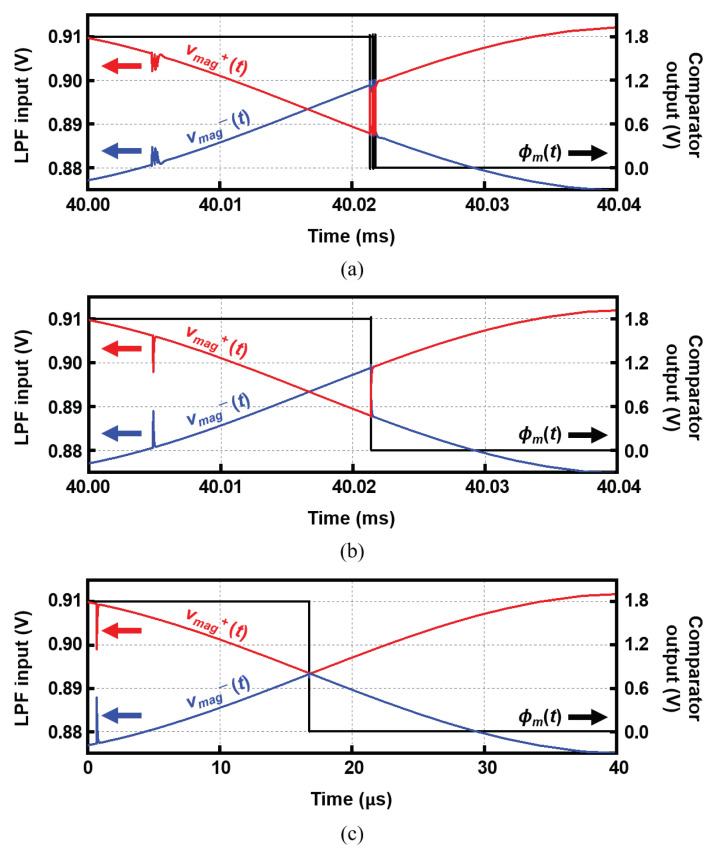
Transient simulation results of the output signals of the chopper and the comparator ($v_{mag}^{+}\left(t\right)$, $v_{mag}^{-}\left(t\right)$, and $\phi_{m}\left(t\right)$) (**a**) without and (**b**) with the buffers. (**c**) The signals with the buffers and offset cancellation by the autozeroing.

Figure 12a–c compares the operation of the chopper and comparator with and without the buffer when $f_{in}=$ 10 kHz, $i_{in}=$ 2 $\mu A_{PP}$ and $A_{IA}=$ 33.6 dB. Figure 12a shows the output signals of the chopper ($v_{mag}^{+}\left(t\right)$ and $v_{mag}^{-}\left(t\right)$) and the comparator ($\phi_{m}\left(t\right)$) when operating without the buffer and the offset cancellation.

The comparator converts the IA outputs of sinusoidal waveform to a square waveform $\phi_{m}\left(t\right)$, and the chopper operates at $\phi_{m}\left(t\right)$, generating the rectified signal ($v_{mag}^{+}\left(t\right)$ and $v_{mag}^{-}\left(t\right)$), which is fed to the LPF (Figure 5). During this chopping operation, sparks are generated and this kick-back noise severely affects the IA outputs. Then, this again returns to the chopper clock, leading to an oscillating output at the comparator, as shown in Figure 12a. When there is no offset in the comparator, this is not a serious problem because the kick-back spark noises occur exactly at the crossing point every time. In contrast, when there is an offset or low-frequency noise at the comparator, it generates a kick-back phenomenon such as that shown in Figure 12a.

To prevent this, a buffer is added between the IA and the choppers, as shown in Figure 5. This isolates the chopper from the comparator inputs (the IA outputs) and breaks the loop, preventing the sparks from returning. Figure 12b plots the signals with the buffers. Even with the offset, a neat square-waveform signal is generated without any kick-back phenomenon.

However, there is still the offset of 15 mV in the case of Figure 12b. Thus, $\phi_{m}\left(t\right)$ is delayed from the exact zero-crossing point of $v_{mag}^{+}\left(t\right)$ and $v_{mag}^{-}\left(t\right)$. Therefore, in the proposed IC, the autozeroing technique operates periodically every 10 ms to remove the offset effectively. Figure 12c shows the signals after the offset cancellation. The transition point of $\phi_{m}\left(t\right)$ and the crossing point of $v_{mag}^{+}\left(t\right)$ and $v_{mag}^{-}\left(t\right)$ are almost coincident. This shows that the autozeroing technique successfully removes the offset.

### 4.3. Settling-Time Reduction

For the $V_{mag}$, $V_{mag,r}$, and $V_{real}$ to reach the final DC value after LPF, the settling time is required. This consists of rising time as slew rate and the linear settling time. Since the slew rate is due to the limitations in the current system, $V_{mag,r}$ and $V_{mag}$ rise with the same slope. On the other hand, the linear settling is related to the cut-off frequency ($f_{c}$) in proportion to the final value as below:

(17) $$\begin{array}{cc} V_{mag}\left(t\right) & =V_{mag}\left(1-e^{-2\pi f_{c}t}\right)\\ V_{mag,r}\left(t\right) & =V_{mag,r}\left(1-e^{-2\pi f_{c}t}\right). \end{array}$$

So, when it is measured as a ratio, the cut-off-frequency-dependent term ($1-e^{-2\pi f_{c}t}$) is canceled out, and accurate data can be obtained immediately after the slew section.

**Figure 13:**
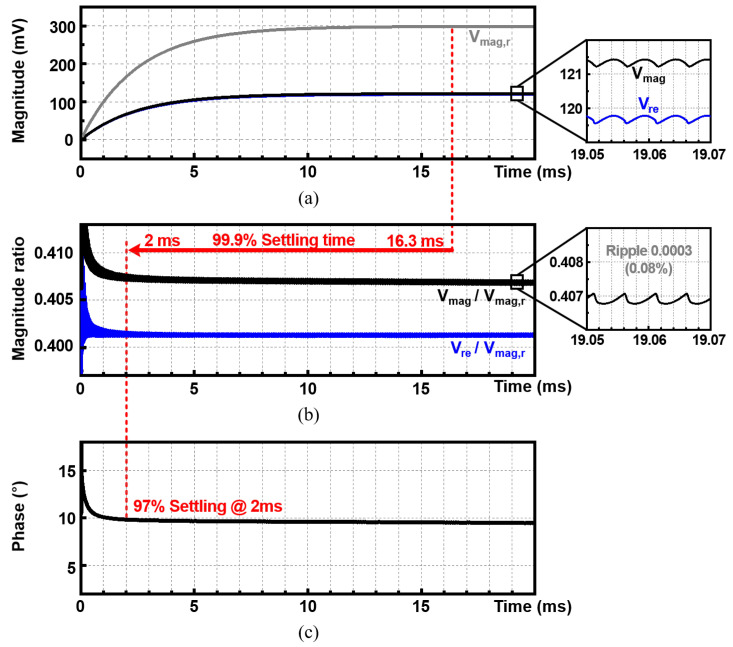
Transient simulated waveforms of (**a**) the reference magnitude output ($V_{mag,r}$), measured magnitude output ($V_{mag}$), real output ($V_{re}$), (**b**) the ratio of them ($V_{mag}/V_{mag,r}$ and $V_{re}/V_{mag,r}$), and (**c**) the phase results, when $f_{in}=$ 100 kHz, $i_{in}=$ 4 μ$A_{pp}$, and $A_{IA}=$ 48.7 dB.

Figure 13 shows (a) the final voltage outputs, $V_{mag,r}$, $V_{mag}$, $V_{re}$, (b) the ratios, $V_{mag}/V_{mag,r}$, $V_{re}/V_{mag,r}$, and (c) the phase result, when $f_{in}=$ 100 kHz, $i_{in}=$ 4 μ$A_{pp}$, and $A_{IA}=$ 48.7 dB. Each final voltage output ($V_{mag,r}$, $V_{mag}$, and $V_{re}$) takes 16.3 ms to reach 99.9% accuracy. In contrast, for the ratio ($V_{mag}/V_{mag,r}$ and $V_{re}/V_{mag,r}$), the settling time to achieve the same 99.9% accuracy is only 2 ms, which is an 87.7% reduction from 16.3 ms. Note that $V_{mag}/V_{mag,r}$ and $V_{re}/V_{mag,r}$ reach this point with only 7.3% and 5% errors even at 350 μs, respectively. Using Equation (16), the phase result is calculated from the ratio value. As shown in Figure 13c, it also settles much faster than the voltage outputs.

In addition, as shown in the inset of Figure 13b, the ripple is only 0.08% of the final value. This means that the LPF sufficiently removes the high-frequency components, which are modulated up by the chopper. As shown using the proposed ratio detection, much shorter settling times can be achieved with very small ripples.

### 4.4. Impedance Readout Resuylts

**Figure 14:**
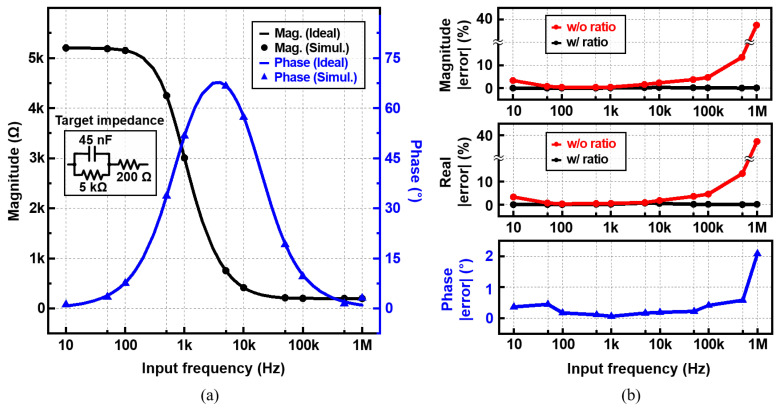
Simulated (**a**) magnitude, phase, and (**b**) the errors obtained using the proposed measurement IC over the input frequency ($f_{in}$).

Figure 14 shows $|Z_{m}|$ and $\theta_{m}$ results along with the errors. The target impedance shown in the inset of Figure 14a is used. In Figure 14a, the theoretical impedance values are shown as solid lines, and the simulation results are indicated as circles and triangles. The readout results well match with the theoretical values over the entire frequency range of 10 Hz–1 MHz. The magnitude, real and phase errors are shown in Figure 14b. The magnitude error calculated by Equation (5) is shown in red, and the error calculated by Equation (7) is shown in black. Since Equation (5) includes only the $Z_{m}$ Mag-path result, it is greatly affected by the nonlinearity of IA and the delay error term. Thus, the error tends to increase as the frequency increases. As a result, by measuring the magnitude with ratio detection, the maximum $|Z_{m}|$ error is reduced from 37.5% to 0.27%. The $RE(Z_{m})$ error also decreased from 37.3% to 0.68%. The $|Z_{m}|$ and $\theta_{m}$ errors are within ±0.3% and ±2.1° respectively.

**Figure 15:**
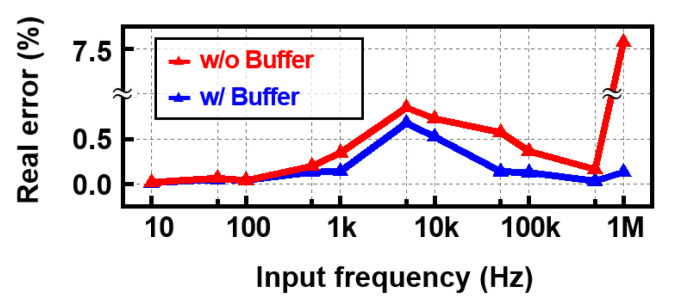
Simulated real error with and without the buffer over the input frequency ($f_{in}$).

Figure 15 shows the effect of buffer on the $RE(Z_{m})$ accuracy. Since the real path has no loop including the chopper input and IA output, the errors of with and without buffer can be compared without kick-back phenomenon. The high-frequency signal has a short period, so the error caused by the comparator delay becomes significant. Thus, the compensation effect by the buffer is remarkable as frequency increases. In addition, since the $RE(Z_{m})$ is in cosine form of $\theta_{m}$, $RE(Z_{m})$ error of 1–10 kHz is larger due to the large $\theta_{m}$.

### 4.5. Performance Summary and Comparison

Table 1 summarizes the performances of the proposed IC and compares them with other state-of-the-art works. A time-stamp IC [34] can cover a 10-times-higher frequency range while consuming 55 times higher power. Other works cover a similar or lower frequency range with much higher power consumption. Therefore, the proposed work achieves one of the best power efficiencies while providing one of the highest $|Z_{m}|$ accuracies and widest frequency ranges.

## 5. Conclusions

We proposed the new impedance measurement IC for EIS to support a wide frequency range. In the proposed IC, the reference resistor and its magnitude-measurement path are implemented to compensate for the delay and nonlinearities of the VCCS and IA. The magnitude and real components are measured as a ratio to a reference magnitude, greatly enhancing the measurement speed by more than eight times. Thanks to this measurement method that significantly reduces the effect from many error sources, the frequency range up to 1 MHz can be supported by using the IA with a small bandwidth of 200 kHz, resulting in total power consumption of only 0.513 mW. Moreover, the autozeroing technique was employed to eliminate the offset, and buffers were used to prevent the kick-back noise and to compensate for the error caused by the comparator delay. According to the simulation results, the real and magnitude accuracies of our proposed IC are 99.7% and 99.4%, respectively, over a wide frequency range of 10 Hz–1 MHz. The corresponding phase error is less than 0.6° up to 500 kHz, and 2.1° up to 1 MHz.

## Figures and Tables

**Table 1:** Performance summary and comparison with state-of-the-art works.

Parameters	AD5933 [27]	IEEE Sensors 2013 [24]	IEEE BioCAS 2016 [32]	ISOCC 2015 [33]	IEEE MWSCAS 2013 [34]	IEEE TBCAS 2020 [35]	This Work
Architecture	I/Q	Polar	Polar	Polar	Polar	Time Stamp	Real/Mag
Phase detection method	−	Integrator	TDC	TDC	TDC	TDC	LPF
Process (μm)	−	0.35	0.25	0.18	0.18	0.18	0.18
Supply voltage (*V*)	3.3	±2.5	2.5	1.8	±0.9	±1.65	1.8
Power consumption (mW)	33.0 ${}^{b}$	21.0 ${}^{b}$	10.3 ${}^{a}$	10.0 ${}^{a}$	28.0 ${}^{a}$	0.684 ${}^{b}$	0.513 ${}^{a}$
Frequency range (Hz)	1–100 k	100–100 k	1 k–2.048 M	100–100 k	100–10 M	10–500 k	10–1 M
Magnitude error (%)	<2 ${}^{b,c}$	<3.5 ${}^{b}$	<1.0 ${}^{a}$	<1.9 ${}^{a}$	<2.5 ${}^{a}$	<2.94 ${}^{b}$	<0.3 ${}^{a}$
Phase (°)	<0.9 ${}^{b,c}$	<3.6 ${}^{b}$	<1.3 ${}^{a}$	<0.2 ${}^{a}$	<2.2 ${}^{a}$	<1.19 ${\%}^{b}$	<0.6 ${}^{a}$ (500 kHz) <2.1 ${}^{a}$ (1 MHz)

${}^{a}$ Simulation results, ${}^{b}$ Measurement results, ${}^{c}$ Estimated from datasheet.
